# Effectiveness of the Gibbs' reflective cycle in humanistic care training for infectious disease nurses: a quasi-experimental study

**DOI:** 10.3389/fpubh.2026.1886603

**Published:** 2026-08-19

**Authors:** Sijia Li, Huiying Chen, Jia Guo, Wen Zhang

**Affiliations:** Nursing Department, Sir Run Run Shaw Hospital, Zhejiang University School of Medicine, Hangzhou, Zhejiang Province, China

**Keywords:** ability, Gibbs' reflection cycle, humanistic care, infectious disease, nursing education

## Abstract

**Aim:**

In infectious disease settings, patients often encounter isolation, stigma, and psychological distress. Traditional humanistic training is frequently lecture-based and lacks structured reflection, limiting its ability to enhance practical caring behaviors. This study aimed to evaluate the effectiveness of the Gibbs' reflective cycle in humanistic care training for nurses in infectious disease departments.

**Methods:**

In 2025, this quasi-experimental study involved 60 registered nurses from an infectious disease department in a tertiary Chinese hospital. Thirty received humanistic training with the Gibbs' reflective cycle, while 30 received conventional training. Outcomes (Caring Behavior Inventory, Reflective Ability Scale) were assessed at baseline and post-training. Paired and independent t-tests were used to compare within- and between-group differences.

**Results:**

After training, the intervention group exhibited significantly higher total and dimension scores on the humanistic care behavior scale compared to their pre-training scores and those of the control group (*P* < 0.05). Similarly, the intervention group's total and dimension scores on the reflective ability scale were also higher than their pre-training scores and those of the control group (*P* < 0.05).

**Conclusion:**

The humanistic training model based on the Gibbs' reflective cycle is associated with significant improvements in the humanistic care behavior and reflective ability of nurses in infectious disease departments. It offers strong support for delivering high-quality nursing services and is worthy of clinical promotion and application.

## Introduction

Modern medicine has been shifting from a purely biomedical model to a biopsychosocial one. Reflecting this transition, the Healthy China 2030 Plan Outline explicitly emphasizes the importance of medical humanistic care. Consequently, humanistic nursing care has become a key indicator of nursing service quality in China.

In practice, humanistic care means respecting patients' life values, personal dignity, and diverse needs throughout diagnosis, treatment, and care. This approach serves as a cornerstone for building harmonious nurse-patient relationships (1). This need is especially pronounced in infectious disease departments, which are dedicated to preventing and treating infectious illnesses. Patients in these departments often have highly transmissible

conditions, complex and fluctuating clinical courses, prolonged treatment periods, and limited social support. These features make them more vulnerable to anxiety, depression, and loneliness than those in general wards, creating a substantially greater demand for humanistic care (2).

Therefore, fostering high-level humanistic competence among healthcare professionals has become an urgent priority in the development of infectious disease specialties (3). Nevertheless, humanistic care training for nurses in infectious disease departments remains insufficient. Common shortcomings include inadequate emphasis on cultivating humanistic qualities, a relatively small proportion of relevant course content, and the lack of systematic, structured training programs (4, 5). These deficiencies not only hinder improvements in nursing service quality but also negatively affect patients' overall care experiences.

Gibbs' Reflective Cycle, introduced by British scholar Graham Gibbs in 1988, is a structured reflection model comprising six stages: description, feelings, evaluation, analysis, conclusion, and action plan. This model guides practitioners through systematic reflection and supports subsequent behavioral improvement (6). It has proven effective in nursing education both domestically and internationally, notably enhancing learners' problem-solving and practical skills (7, 8). Despite its proven value in general nursing education, a significant research gap exists. Prior studies have largely focused on the application of Gibbs' Reflective Cycle in academic settings with nursing students (9, 10). Evidence regarding its systematic integration into continuing professional development programs for registered nurses—particularly those in high-stress, emotionally demanding specialties like infectious disease nursing—remains scarce. Specifically, no study has examined how a structured, multi-component training program built around Gibbs' Reflective Cycle can improve the observable humanistic caring behaviors and reflective abilities of clinical nurses in this field.

Given that humanistic care requires nurses to internalize patient-oriented values and translate them into empathetic practice, the structured and cyclical nature of Gibbs' cycle is particularly well suited to this purpose. It offers a clear framework for deconstructing care encounters, identifying gaps in compassionate response, and formulating actionable improvement plans. Therefore, this study was designed to address the gap in the literature by evaluating a novel training model that systematically integrates Gibbs' Reflective Cycle into humanistic care training for infectious disease nurses, with the aim of providing evidence for a standardized and replicable professional development program.

We hypothesized that: (1) nurses in the intervention group would demonstrate significantly higher levels of humanistic caring behaviors than those in the control group post-training; and (2) nurses in the intervention group would demonstrate significantly higher levels of reflective ability than those in the control group post-training.

## Methods

### Study design

A concurrent controlled quasi-experimental study using two non-equivalent intact ward groups was conducted between April and October 2025.

### Participants

Participants were registered nurses working in the Department of Infectious Diseases at a tertiary Grade A general hospital in Hangzhou, Zhejiang Province, China. The sample size was determined using G^*^Power software for a two-tailed independent *t*-test. Based on previous literature on humanistic care training, a moderate effect size (Cohen's *d* = 0.80) was anticipated. With a significance level (α) of 0.05 and a statistical power (1-β) of 0.80, the required sample size was calculated as 26 participants per group. Accounting for a 15% potential attrition rate, the target enrollment was set at 30 participants per group, resulting in a total sample size of 60. Inclusion criteria were as follows: holding a valid nursing license, being employed full-time in the infectious disease department with specialist nursing qualifications, having at least 1 year of clinical nursing experience in the same department, and being capable of independent clinical care. Participants also needed to be able to attend the entire training programme and complete all data collection. Exclusion criteria included: nursing interns, visiting nurses, or rotational nurses, as well as those unable to participate throughout the study period due to leave or resignation.

Two cohorts were established according to the routine ward-based training arrangements during the study period. The use of intact ward groups was chosen for pragmatic reasons aligned with routine training schedules; however, this non-random allocation may introduce selection bias. To mitigate this risk, baseline characteristics were compared between groups. Thirty nurses from Infectious Disease Ward 1 comprised the intervention cohort, who received a humanistic care workshop based on Gibbs' reflective cycle. Thirty nurses from Ward 2 served as the control cohort, who undertook conventional humanistic care training. To prevent cross-group contamination, the two wards were located on different hospital campuses of the same tertiary hospital, with separate nursing management teams and minimal cross-campus staff movement. Both wards had similar patient acuity and nurse-patient ratios during the study period. This study was approved by the Medical Ethics Committee of Sir Run Run Shaw Hospital (Ethics Approval Number: 2025-0507). All participants provided written informed consent and participated voluntarily. Both cohorts completed a 3-month training program totaling eight contact hours. Training duration, faculty, and other basic resources were identical between the two cohorts; the only difference was the training model implemented in routine clinical practice.

### Intervention

#### Control group

The control group received conventional humanistic care training as delivered in routine ward practice, primarily through didactic lectures combined with case-based explanations. The training consisted of two parts: didactic lectures (4 h) covering core concepts of humanistic care, nurse-patient communication skills, psychological characteristics of patients, and key elements of emotional support; and typical case discussions (4 h) analyzing how humanistic care applies to clinical nursing practice. No systematic reflective exercises or scenario-based simulations were included in this training.

#### Intervention group

The experimental group participated in the routine ward-based humanistic care workshop grounded in Gibbs' Reflective Cycle. The program consisted of three sequential components: structured analysis of typical clinical cases, standardized scenario-based simulations, and structured reflective journal writing with sharing. The goal was to foster deep, systematic reflection on common humanistic care situations in infectious disease settings, helping nurses internalize humanistic care principles and apply them in practice. The training team included a head nurse with over 10 years of experience in infectious disease nursing management, a full-time nurse educator, and a member of the hospital's Humanistic Care Committee. All team members received prior training in Gibbs' reflective cycle and facilitation techniques, and passed an assessment before teaching. To ensure intervention fidelity, a standardized facilitator guide was used, each session was observed by a non-participating researcher using a structured fidelity checklist, and biweekly calibration meetings were held; over 90% of core content elements were delivered as planned across all sessions.

### Structured analysis of typical clinical cases (3 h)

Typical humanistic care cases from infectious disease practice were used, including: psychological support for a malaria patient under isolation, addressing stigma in a hepatitis B patient, end-of-life care for a patient with terminal liver disease, and psychological counseling for family members of infectious disease patients. The intervention group was divided into three subgroups, with 10 nurses in each. Each subgroup selected one case and, under the guidance of the trainers, worked through the six stages of Gibbs' cycle: description, feelings, evaluation, analysis, conclusion, and action plan. A representative from each subgroup presented the group's analysis. Trainers then provided feedback on the depth of reflection and the accuracy of problem identification, helping nurses strengthen their humanistic care perspective.

### Standardized scenario-based simulation and immediate reflection (3 h)

Several common humanistic care conflict scenarios were designed for the infectious disease setting, including: a patient refusing treatment due to resistance to isolation, family members distressed by concerns about prognosis, communication difficulties between young nurses and older infectious disease patients, and psychological issues arising from stigma and fear of transmission. Participants were randomly assigned roles—patient, family member, primary nurse, or head nurse—and acted out realistic clinical interactions. Each simulation lasted approximately 15 min. Afterwards, participants engaged in immediate structured reflection using Gibbs' six-stage model, sharing their role-based experiences and identifying strengths and areas for improvement. Other group members provided feedback, and trainers helped participants analyze root causes and identify optimal communication and humanistic care strategies for infectious disease practice.

### Structured reflective journal writing and sharing (2 h)

During the training period, each nurse wrote one structured reflective journal per month about a humanistic care event from clinical practice, such as successfully easing a patient's anxiety, a nurse-patient conflict caused by poor communication, or challenges in delivering humanistic care. The journals followed Gibbs' six stages, with an emphasis on the depth of problem analysis and the feasibility of proposed improvements. Two journal-sharing sessions were held, one at the midpoint and one at the end of the training. During these sessions, representative journals were discussed as a group. Trainers provided targeted feedback on reflection depth, problem analysis, and action plans, helping nurses consolidate their learning and build a lasting habit of reflective practice. The specific application procedures of Gibbs' reflective cycle in the humanistic care training for infectious disease nurses are presented in Table 1.

**Table 1:** Application of Gibbs' reflection cycle model in humanistic care training for infectious disease nurses.

Stage	Core content	Key teaching guidance
Description	Objectively present the time, place, people involved, event process, and specific nursing actions, avoiding subjective judgment	Guide nurses to describe key details accurately. Example: “The patient refused to take medication because of concerns about antiviral side effects. I briefly explained why the medication was necessary but did not explore their concerns in depth. The patient remained non-adherent”
Feelings	Express one's own thoughts and emotional responses to the event, and try to understand the patient's and family's feelings and needs	Encourage nurses to be honest about genuine emotions (e.g., helplessness, self-blame, relief). Use prompts like, “How would you feel if you were facing isolation?” to foster empathy
Evaluation	Objectively assess the strengths and weaknesses of nursing actions from professional, communication, humanistic, and outcome perspectives	Help nurses focus on how their actions affected the patient. Example: “I noticed the patient's emotional change promptly, which is good, but I did not explore the real reason for treatment refusal. My communication lacked depth, and the outcome was not achieved”
Analysis	Analyse the causes of identified problems by integrating infectious disease characteristics, clinical knowledge, humanistic theories, and communication skills	Help nurses distinguish subjective factors (e.g., limited humanistic awareness, rigid communication, lack of specialty knowledge) from objective ones (e.g., low health literacy, poor social support, severe stigma). Example: “The patient's refusal of isolation was mainly due to misunderstanding about transmission routes and fear of discrimination. I failed to provide targeted health education or psychological support”
Conclusion	Summarize lessons learned and develop new insights and approaches to humanistic care in infectious disease practice	Guide nurses to identify practical lessons that can be applied elsewhere. Example: “When caring for isolated infectious disease patients, one should first show empathy to ease negative emotions, then provide tailored health education based on clinical knowledge, while involving family members for emotional support”
Action plan	Develop specific, actionable, and measurable improvement strategies, clarifying directions and goals for future clinical practice	Ask nurses to make plans based on their own practice. Example: “For similar patients in the future, I will complete health education on infectious disease knowledge within 24 h, communicate with the patient and family at least twice a week, and assess psychological status promptly to offer support”

### Outcome measures

#### Caring behavior inventory (CBI)

The Caring Behavior Inventory (CBI) was developed by Wolf (11) based on Watson's Theory of Human Caring. It was later revised by Wu (12) and adapted to Chinese by Da (13). The scale measures nurses' caring behaviors across three dimensions: Support and Guarantee (9 items), Knowledge and Skills (5 items), and Respect and Contact (10 items). It consists of 24 items, each rated on a six-point Likert scale from 1 (never) to 6 (always). Total scores range from 24 to 144, with higher scores reflecting stronger caring behaviors. The scale demonstrates good psychometric properties: Cronbach's alpha is 0.959, and the content validity index (CVI) is 1.00.

#### Reflective ability scale for clinical nurses

This scale was developed by Nishimoto (14) and later adapted to Chinese by Shao (15). It measures clinical nurses' reflective ability across three dimensions: Reviewing Nursing Practice (8 items), Reflecting on Nursing Practice (6 items), and Extending Nursing Practice (5 items). The instrument consists of 19 items on a six-point Likert scale ranging from 1 (strongly disagree) to 6 (strongly agree). Total scores range from 19 to 114, with higher scores indicating greater reflective ability. The scale has sound psychometric properties, with a Cronbach's alpha of 0.901 and a CVI of 0.947.

The evaluations were conducted by two supervising instructors from the infectious disease department. To minimize potential bias, although the evaluators were aware of the different training modules the nurses had undergone as part of routine ward practice, they were kept unaware of the specific academic purpose of comparing the two groups and the subsequent data analysis. They were instructed to administer the scales in a standardized, impartial manner and were blinded to the participants' baseline scores. This partial blinding procedure was implemented to minimize potential bias.

#### Data collection and statistical analysis

Data were collected at two time points: before training (T0) and within 1 week after training (T1). The two scales were electronically distributed to nurses in both groups. Before distribution, the researchers explained the purpose, completion instructions, and confidentiality principles. All questionnaire items were mandatory, and each questionnaire took approximately 5–10 min to complete. All 60 distributed questionnaires were returned, resulting in a response rate of 100%. After excluding those with obvious logical inconsistencies or identical responses across all items, 60 valid questionnaires remained, yielding an effective response rate of 100%.

Statistical analyses were performed using SPSS version 26.0. Normality of continuous variables was assessed using the Shapiro–Wilk test and visual inspection of Q-Q plots, and homogeneity of variances was evaluated using Levene's test. Normally distributed continuous data are presented as mean ± standard deviation (SD), and categorical variables as frequencies and percentages, with chi-square (χ^2^) tests used to examine between-group differences. When assumptions were violated, the non-parametric Mann–Whitney U test or Wilcoxon signed-rank test was employed as a sensitivity analysis; results were consistent with those from the parametric tests reported. Given the two-time-point design and the specific research questions focusing on within-group changes and between-group differences at post-test, paired and independent *t*-tests were considered appropriate. As baseline characteristics were comparable between the two groups, no adjustment for baseline covariates was performed in the primary analysis. A two-tailed significance level of α = 0.05 was set, and *P* < 0.05 was considered statistically significant.

## Results

### General characteristics of the participants

All 60 enrolled participants completed the full training program and all assessments, with no dropouts. No statistically significant differences were found between the two groups in baseline demographic and clinical characteristics, including age, years of clinical experience, marital status, and professional title (all *P* > 0.05), confirming the comparability of the two groups at baseline. A detailed summary of these characteristics is provided in Table 2.

**Table 2:** Comparison of general characteristics between the two groups.

General information	Intervention group (*n* = 30)	Control group (*n* = 30)	*t*/χ^2^	*P*
Age (years)	29.66 ± 5.24	29.53 ± 5.24	0.08	0.94
Clinical experience (years)	7.28 ± 6.21	6.60 ± 5.18	0.45	0.65
Marital status				
Unmarried	14	18	1.07	0.30
Married	16	12		
Professional title				
Junior	16	18	0.27	0.60
Senior	14	12		

### Comparison of caring behavior inventory (CBI) scores between the two groups

After the humanistic care training based on Gibbs' reflective cycle, nurses in the intervention group scored higher on all CBI dimensions than they did before the training and than those in the control group. All these differences were statistically significant (*P* < 0.05). Detailed results are shown in Table 3.

**Table 3:** Comparison of CBI scores between the two groups.

Variables		Intervention group	Control group	*t*	*P*	Cohen's d
Support and guarantee	Before	47.03 ± 4.99	48.53 ± 6.52	−1.00	0.32	0.26
	After	50.34 ± 4.15^*^	47.40 ± 5.16	2.41	0.02	0.63
Knowledge and skill	Before	26.30 ± 2.55	27.00 ± 3.25	−0.93	0.36	0.24
	After	27.69 ± 2.30^*^	26.00 ± 2.67	2.60	0.01	0.68
Respect and contact	Before	45.70 ± 6.89	46.40 ± 7.59	−0.37	0.71	0.10
	After	51.55 ± 6.35^*^	48.23 ± 5.60	2.13	0.04	0.56
Total score	Before	119.03 ± 12.71	121.93 ± 16.37	−0.78	0.45	0.20
	After	129.59 ± 11.33^*^	121.63 ± 12.63	2.54	0.01	0.67

^*^P < 0.05 compared with baseline within the same group. Cohen's d represents effect size for between-group comparisons.

### Comparison of reflective ability scale scores between the two groups

After the Gibbs-based humanistic care training, the intervention group exhibited significantly higher scores on all dimensions of the Reflective Ability Scale for Clinical Nurses compared to both the baseline and the control group. These differences were statistically significant (all *P* < 0.05). Table 4 presents the detailed results.

**Table 4:** Comparison of reflective ability scale scores between the two groups.

Variables		Intervention group	Control group	*t*	*P*	Cohen's d
Recall Nursing Practice	Before	38.07 ± 5.23	40.47 ± 5.96	−1.66	0.10	0.43
	After	41.03 ± 4.84^*^	37.93 ± 4.10	2.66	0.01	0.69
Reflect on nursing practice	Before	28.77 ± 3.40	29.23 ± 3.88	−0.50	0.65	0.13
	After	30.93 ± 4.04^*^	28.77 ± 2.01	2.59	0.01	0.68
Expand nursing practice	Before	23.43 ± 3.95	24.77 ± 3.51	−1.38	0.17	0.36
	After	25.79 ± 3.22^*^	24.00 ± 2.05	2.54	0.01	0.66
Total score	Before	90.27 ± 12.53	94.47 ± 12.84	−1.28	0.21	0.33
	After	97.76 ± 11.65^*^	90.70 ± 7.54	2.75	0.01	0.72

^*^P < 0.05 compared with baseline within the same group. Cohen's d represents the effect size for between-group comparisons.

## Discussion

This quasi-experimental study compared the effects of conventional humanistic training and a Gibbs' reflective cycle-based workshop on the humanistic caring behaviors and reflective abilities of nurses in infectious disease departments. Our findings revealed that Gibbs' Reflective Cycle offers significant advantages in enhancing both outcomes. Specifically, nurses in the intervention group scored significantly higher than those in the control group on both the Caring Behavior Inventory (CBI)—particularly in the dimensions of 'support and guarantee', 'knowledge and skills', and 'respect and contact'—and the Reflective Ability Scale for Clinical Nurses. Beyond statistical significance, the observed effect sizes (Cohen's *d* = 0.67 for CBI total and *d* = 0.72 for reflective ability total) indicate moderate-to-large practical effects, suggesting practical relevance in clinical training. In educational terms, these improvements suggest that the Gibbs' Reflective Cycle enabled nurses to meaningfully translate humanistic concepts into actionable clinical behaviors. These findings indicate that this structured, reflective learning approach effectively addresses the shortcomings of traditional lecture-based methods and improves nurses' overall humanistic care competence, which may positively influence the quality of nursing services in infectious disease departments. Overall, this suggests that the Gibbs' Reflective Cycle not only produced a measurable difference but also one that is educationally meaningful.

### Gibbs' reflective cycle-based training improves humanistic caring behaviors in infectious disease nurses

Patients in infectious disease departments often have unique psychological needs due to the contagious nature of their illnesses and the risk of social stigma, which makes them more susceptible to anxiety, depression, and isolation. Conventional humanistic care training mainly relies on lectures, with few immersive clinical experiences or systematic reflection components, making it difficult for nurses to transform abstract humanistic concepts into concrete nursing actions. In this study, after the Gibbs-based training, nurses in the intervention group scored higher on both the total and dimension scores of the Caring Behavior Inventory (CBI). This finding is consistent with Zhang (16), who found that reflective learning methods not only enhance professional knowledge and skills but also assist nurses in applying humanistic care in clinical practice.

Gibbs' reflective cycle provides a clear, step-by-step approach to delivering humanistic care through its six stages: description, feelings, evaluation, analysis, conclusion, and action plan. Unlike traditional didactic training, which presents humanistic principles as abstract knowledge to be memorized, the Gibbs' cycle embeds these principles within the nurse's own clinical experiences. In the description and feelings stages, nurses objectively recount clinical events and attempt to view things from the patient's perspective. This helps them better understand what patients are experiencing and enhances empathy. The evaluation and analysis stages guide nurses to identify gaps in their humanistic care practices and explore the reasons for these gaps in infectious disease nursing. Finally, the conclusion and action plan stages translate reflective insights into concrete improvement steps, creating a cycle of practice, reflection, improvement, and renewed practice. This structured approach not only facilitates the internalization of humanistic values but also cultivates the habit of critically evaluating one's own performance.

This approach aligns with Zhou's case-based teaching method for infectious disease nursing postgraduates (17), which also emphasizes active learning and deep reflection in clinical or simulated settings. It is further supported by broader international evidence. Polat Dünya et al. (10) found that nursing students using Gibbs' Reflective Cycle with authentic patient narratives developed greater awareness of the psychosocial aspects of illness, stronger empathy and ethical sensitivity, and a clearer understanding of the theory-practice gap—all outcomes that mirror those observed in our sample of registered nurses. The fact that similar benefits emerged in both student and practicing nurse populations suggests that Gibbs' cycle is a flexible tool capable of supporting professional growth across different career stages. Taken together, these findings indicate that Gibbs' Reflective Cycle helps nurses transition from passively receiving knowledge to actively evaluating their own practice, thereby facilitating the meaningful integration of humanistic care into daily clinical work.

### Gibbs' reflective cycle-based training improves clinical reflective ability in infectious disease nurses

Reflective ability is fundamental to nurses' ongoing professional development and is key to improving the quality of nursing care. In this study, the intervention group achieved higher total and dimension scores on the Reflective Ability Scale for Clinical Nurses, suggesting that Gibbs' reflective cycle effectively addresses a common drawback of traditional training—namely, its insufficient and unstructured reflective guidance. This finding is consistent with Wu's observation that the overall reflective ability of clinical nurses remains at a moderate to low level, and that participation in reflective training is a major influencing factor (18). Similarly, Zhang combined anchored instruction with Gibbs' reflective cycle in the training of operating theater specialist nurses and reported significant improvements in both theoretical knowledge and practical performance, attributing this success to the structured reflection process that enables nurses to systematically review, analyze, and plan their clinical work (19).

In the present study, nurses engaged in the six stages of Gibbs' cycle through three complementary training activities: structured analysis of typical clinical cases, standardized scenario-based simulations with immediate reflection, and structured reflective journal writing and sharing. During the description and feelings stages, nurses learned to recount events objectively and acknowledge their emotional responses, while the evaluation and analysis stages encouraged critical thinking and facilitated the identification of root causes. The conclusion and action plan stages then translated reflective insights into concrete strategies for future practice. Importantly, the repeated application of this structured framework across multiple modalities likely reinforced the habit of systematic reflection, transforming it from a one-time exercise into an automatic component of clinical reasoning. This process not only strengthened nurses' ability to review and reflect on past experiences but also enhanced their capacity to plan and extend future nursing practice—competencies that are particularly valuable for infectious disease nurses, who frequently encounter complex and emotionally charged situations requiring continuous quality improvement.

The broader applicability of Gibbs' Reflective Cycle in fostering professional competencies is further supported by Hemati et al. (20), who integrated structured reflective discussions using the same six-stage model into a spiritual care education program and observed sustained improvements in nurses' professional competence. Their findings suggest that the reflective mechanism activated by Gibbs' cycle may transcend specific content domains, enhancing clinical reasoning and self-evaluation skills across diverse nursing contexts.

### Advantages of the Gibbs-based humanistic training model in infectious disease nursing

The humanistic care workshop developed in this study, centered around Gibbs' reflective cycle, is well-suited to the specific requirements of infectious disease nursing and the learning preferences of nurses. It offers several practical advantages.

First, the training content is both relevant and transferable. The training cases and scenarios were directly derived from real clinical situations in the infectious disease department, focusing on the actual humanistic care needs of patients and the challenges nurses encounter. This aligns with Yuan's view that teaching cases should be drawn from authentic clinical practice (21). Importantly, the core challenges addressed—patient isolation, stigma, communication barriers, and emotional distress—are universal in infectious disease nursing across the globe. Therefore, the case-based reflective approach developed in this study can be adapted to similar settings in other countries, providing a replicable model for enhancing humanistic care globally.

Second, the model is easy to implement and resource-efficient. The six-stage framework of Gibbs' reflective cycle is clear and logical, requiring no complex theoretical background or expensive equipment. Nurses can quickly understand, master, and apply it in clinical work shortly after training. This low-cost, high-impact characteristic makes the model particularly suitable for resource-limited settings, where advanced simulation facilities or lengthy training programs may not be feasible. International educators seeking affordable yet effective strategies to enhance humanistic care may find this approach highly applicable. However, effective facilitation remains important to ensure deep rather than superficial reflection.

Third, the training adopts diverse formats. By combining structured case analysis, standardized scenario-based simulations, and reflective journal sharing, this approach moves beyond the traditional lecture-based model. The variety maintains nurses' engagement and encourages active participation. These methods are widely recognized in international nursing education as effective pedagogies for promoting reflective practice and clinical reasoning. Thus, the study contributes to the growing body of international evidence supporting the integration of active learning strategies into humanistic care training.

Fourth, the training effects are likely to be sustained, thereby addressing a common challenge in continuing professional development on a global scale. Ongoing reflective journal writing and group sharing facilitate the development of a lasting habit of reflection, enabling the transfer of knowledge and skills into long-term clinical practice (22). Importantly, this model does not replace traditional humanistic training but rather serves as a complementary component. When integrated with active learning methods such as case-based teaching and simulation, it reinforces and deepens the impact of multiple educational approaches (23, 24). Given the global threat posed by emerging infectious diseases (e.g., COVID-19, Ebola, Mpox), the program provides an evidence-based and transferable framework for humanistic care training, which can inform nursing education and policy at the international level. Future efforts may draw upon the concept of caring leadership to cultivate a supportive departmental culture that encourages the enduring adoption and reinforcement of humanistic caring behaviors (25).

## Limitations

Several limitations should be acknowledged. First, the quasi-experimental design with ward-based cohort allocation, while pragmatically aligned with routine training schedules, introduces a risk of selection bias. Although baseline characteristics were comparable between groups, the lack of random allocation precludes the exclusion of unmeasured confounding. Second, the single-center setting and sample size of 60 constrain the generalizability of these findings to primary care or low-resource settings. Third, outcomes were measured only immediately post-training (within 1 week), leaving the long-term sustainability of the observed improvements unknown; future studies should incorporate extended follow-up. Fourth, the exclusive reliance on self-report instruments may introduce social desirability and recall biases, and the heightened attention received by the intervention group raises the possibility of a Hawthorne effect, whereby improvements may partially reflect the novelty of the intervention rather than the reflective cycle itself. The absence of objective behavioral assessments or patient-reported outcomes (e.g., satisfaction surveys, dispute frequency) limits the ability to verify that enhanced nurse competencies translate into tangible patient-level benefits. Finally, although evaluators were blinded to baseline scores and the study's academic purpose, they were aware of the distinct training modules as part of routine practice, constituting only partial blinding. Future multicenter randomized controlled trials incorporating extended follow-up, objective behavioral measures, and patient-centered outcomes are warranted to validate and extend these findings.

## Conclusion

In conclusion, this study provides empirical evidence that a humanistic care workshop structured around Gibbs' Reflective Cycle leads to meaningful improvements in both humanistic caring behaviors and clinical reflective ability among infectious disease nurses. By translating abstract humanistic values into actionable clinical competencies through structured reflection, this approach addresses a critical gap in continuing professional development for nurses in high-stress, emotionally demanding specialties. The model is practical, resource-efficient, and adaptable to various clinical settings, offering a promising strategy for enhancing humanistic care quality in infectious disease nursing. We recommend that nursing educators and clinical managers consider integrating structured reflective frameworks into routine training, while acknowledging that future research is needed to explore their long-term sustainability and impact on patient outcomes.

## Data Availability

The raw data supporting the conclusions of this article will be made available by the authors, without undue reservation.
